# Mutations underlying 3-Hydroxy-3-Methylglutaryl CoA Lyase deficiency in the Saudi population

**DOI:** 10.1186/1471-2350-7-86

**Published:** 2006-12-16

**Authors:** Moeenaldeen Al-Sayed, Faiqa Imtiaz, Osama A Alsmadi, Mohammed S Rashed, Brian F Meyer

**Affiliations:** 1 Department of Medical Genetics, King Faisal Specialist Hospital & Research Centre, Riyadh, Saudi Arabia; 2 Arabian Diagnostics Laboratory, King Faisal Specialist Hospital & Research Centre, PO Box 3354, Riyadh 11211, Saudi Arabia; 3 National Laboratory for Newborn Screening, King Faisal Specialist Hospital & Research Centre, PO Box 3354, Riyadh 11211, Saudi Arabia

## Abstract

**Background:**

3-Hydroxy-3-Methylglutaric aciduria (3HMG, McKusick: 246450) is an autosomal recessive branched chain organic aciduria caused by deficiency of the enzyme 3-Hydroxy-3-Methylglutaryl CoA lyase (HL, HMGCL, EC 4.1.3.4). HL is encoded by *HMGCL *gene and many mutations have been reported. 3HMG is commonly observed in Saudi Arabia.

**Methods:**

We utilized Whole Genome Amplification (WGA), PCR and direct sequencing to identify mutations underlying 3HMG in the Saudi population. Two patients from two unrelated families and thirty-four 3HMG positive dried blood spots (DBS) were included.

**Results:**

We detected the common missense mutation R41Q in 89% of the tested alleles (64 alleles). 2 alleles carried the frame shift mutation F305fs (-2) and the last two alleles had a novel splice site donor IVS6+1G>A mutation which was confirmed by its absence in more than 100 chromosomes from the normal population. All mutations were present in a homozygous state, reflecting extensive consanguinity. The high frequency of R41Q is consistent with a founder effect. Together the three mutations described account for >94% of the pathogenic mutations underlying 3HMG in Saudi Arabia.

**Conclusion:**

Our study provides the most extensive genotype analysis on 3HMG patients from Saudi Arabia. Our findings have direct implications on rapid molecular diagnosis, prenatal and pre-implantation diagnosis and population based prevention programs directed towards 3HMG.

## Background

3-Hydroxy-3-Methylglutaric aciduria (3HMG, McKusick 246450) is an autosomal recessive branched chain organic aciduria caused by deficiency of the enzyme 3-Hydroxy-3-Methylglutaryl CoA lyase (HL, HMGCL, EC 4.1.3.4). This mitochondrial enzyme catalyzes the last step of both leucine degradation and ketogenesis[1]. Patients present in the neonatal or infantile period with severe hypoketotic hypoglycemia, metabolic acidosis, hyperammonemia, vomiting and hypotonia [2]. Untreated, this may progress rapidly to coma and death or may result in permanent neurological damage. With treatment, many patients do well although recurrent metabolic decompensation continues to occur especially with prolonged fasting and inter-current infections. Although a rare disease in Europe and Japan, it is reported to be common in Saudi Arabia and Portugal [3-5].

Rapid biochemical diagnosis by blood acylcarnitine analysis on DBS using tandem mass spectrometry reveals elevation of 3-methylglutarylcarnitine and 3-hydroxyisovalerylcarnitine [6]. Urine analysis by gas chromatography mass spectrometry reveals the presence of 3-hydroxy-3-methylglutaric, 3-methylglutaconic and 3-hydroxyisovaleric acids [7,8]. HL can also be measured in various tissues including lymphocytes and fibroblasts [9,10].

The human *HMGCL *cDNA[11] and gene were cloned and localized to chromosome 1p36.11. It is composed of nine exons [12,13]. Many mutations have been reported in various ethnic groups [4,5,11,13-17]. Two mutations "R41Q and F305fs (-2)" have been previously identified in eight patients from Saudi Arabia [18].

In this paper we report our findings on molecular analysis of 72 alleles from Saudi Arabia.

## Methods

### Patients

Two patients from two apparently unrelated families were included after informed consent was obtained. The diagnosis was made by the characteristic blood acylcarnitine and urine organic acid profiles. A further thirty-four 3HMG positive DBS based on acylcarnitine analysis and confirmed by urine organic acid analysis were obtained from the National Laboratory for Newborn Screening. The samples were referred to the laboratory from sick infants where a metabolic disorder was suspected. No additional clinical details are available on these patients. Waiver of informed consent was provided by the Institutional Review Board of the King Faisal Specialist Hospital and Research Centre, on the basis of anonymization of all study samples.

### Sample collection and DNA extraction

DNA was prepared from peripheral blood from 3HMG positive dried bloodspots. Briefly a sample 2 mm in diameter was punched out from each dried blood spot and placed in a 96-well plate. The 2 mm discs were re-hydrated with 10 μl of phosphate-buffered saline (PBS) at room temperature prior to DNA extraction and amplification. Extraction and amplification were carried out as described for the REPLI-g TM kit (Molecular Staging Inc., New Haven, CT, USA) with minor modifications. Extractions were assembled on ice by adding 10 μl of cell lysis solution (400 mM KOH, 10 mM EDTA at pH 8.0, and 50 mM dithiothreitol), mixed briefly with the PBS-soaked discs and incubated for 10 min. Then 10 μl of neutralization solution (800 mM Tris-hydrochloride) was added and mixed gently by pipetting. This lysate mixture (2 μl) was then used as template per 10 μl of whole genome amplification (WGA) reaction.

### Whole genome amplification (WGA)

WGA [19] was performed in a total volume of 10 μl. Amplification reactions were assembled in 96-well plates by mixing 2.5 μl 4× buffer, 5.4 μl ddH_2_O, 0.1 μl ø29 DNA polymerase, and 2 μl of the lysate mixture. Reactions were incubated at 30°C for 16 h and terminated by heating to 65°C for 3 min. The amplification reactions were diluted 50 fold in ddH_2_O (DNA concentration ~10 ng/μl) and dilutions used in PCR amplification and direct sequencing.

### PCR amplification of WGA DNA

For sequence analysis WGA generated DNA was amplified by PCR using intronic primers designed to amplify the 9 coding exons of the *HMGCL *gene (primer sequences available on request). PCR was performed using the DNA Engine PTC-200 (MJ Research; Watertown, MA, USA;[20]) in a final volume of 20 μl containing approximately 10 ng of genomic DNA, 50 mM KCl, 10 mM Tris-HCl (pH 8.3), 1.5 mM MgCl2, 100 μM deoxyribonucleotide triphosphates (dNTPs), 1 unit of Qiagen (Valencia CA, USA) HotStar Taq polymerase, and 50 ng of each primer. Thermocycling consisted of an initial denaturation at 95°C for 15 min followed by 35 cycles of PCR. Each cycle of PCR consisted of denaturation at 94°C for 60 s, annealing at 62–68°C for 60 s and extension at 72°C for 60 s. A final extension step of 10 min at 72°C was added.

### Mutation detection

Sequencing reactions were performed using an Amersham ET Dye Terminator Cycle Sequencing Kit (Amersham Biosciences; Piscataway, NJ, USA [21]) following the manufacturers instructions. Sequencing reactions were desalted and unincorporated nucleotides removed using ethanol precipitation and re-suspended in a formamide EDTA solution for injection on a MegaBACE 1000 DNA Analysis System (Molecular Dynamics; Sunnyvale, CA, USA). Sequence analysis was performed using the SeqManII module of the Lasergene (DNA Star Inc. WI, USA) software package, then compared to the reference GenBank sequence (accession no. AF376770). Numbering commenced with the A of the ATG initiation codon as +1.

### Genotyping

Genotyping of the marker D1S2620 (<0.5 cM from *HMGCL*) was performed using fluorescently labeled primers (5'-FAM-AAGAGTTGTCCAACCAAATTG-3' and 5'-GAATCTGGGATGGGATGT-3') for PCR amplification (essentially as described above) of alleles which were identified following capillary electrophoresis on a Megabace 1000 and fragment size analysis using Genetic Profiler software (Amersham, Sunnyvale, CA). Alleles were assigned alphabetically following genotyping of patient samples and over 100 chromosomes from the normal population.

### URLs

We used the Ensembl website [22] to obtain exon and intron boundaries, the reference GenBank cDNA sequence, and Primer3 to design appropriate primers [23].

## Results

**Figure 1:**
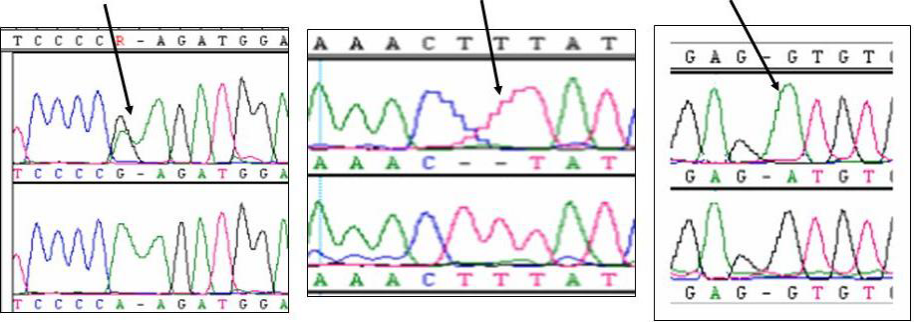
Sequence analysis of PCR-amplified fragments of the *HMGCL *gene of patients and controls. *A*: The arrow indicates a heterozygous R41Q (122 G>A) mutation in a carrier of 3HMG; *B*: The position of the arrow indicates a homozygous F305fs (-2) (-TT) mutation in an affected 3HMG patient and *C*: The arrow indicates a homozygous IVS6+1G>A mutation in an affected 3HMG patient.

Three pathogenic mutations were identified in 72 alleles examined from 3HMG positive DBS. 89% of the alleles (64) were positive for the missense R41Q (122G>A) mutation. One patient was homozygous for the previously reported frame shift mutation F305fs(-2) and another homozygous for a novel splice site donor IVS6+1GA mutation (Figure 1). Demonstration of the pathogenicity of this mutation was not possible as all samples in this retrospective study were anonymized as a condition for IRB approval. This mutation was confirmed by its absence in more than100 chromosomes from the normal population. No mutation was identified in four remaining alleles. Together the three mutations account for 95% of the pathogenic mutations underlying HL deficiency in our Saudi Arabian patients.

All individuals that were homozygous for the R41Q mutation had a B/B genotype at the D1S2620 microsatellite marker on chromosome 1. The incidence of the B allele in the normal population was 0.46, which is statistically significant relative to the 100% incidence in patients with 3HMG deficiency who were homozygous for the R41Q mutation (p = 0.002). This is consistent with R41Q being a founder mutation in the Saudi Arabian population.

## Discussion

In contrast to Europe and Japan, 3HMG is commonly seen in Saudi Arabia [3]. We undertook the task of identifying the underlying mutation in our patients for several reasons. Whilst newborn screening programs are useful for early detection and intervention to reduce morbidity and mortality, they are based upon biochemical profiling which is unable to reliably detect carrier status. Identification of mutations underlying the majority of 3HMG cases in the Saudi population provides opportunities for prevention through socially accepted procedures such as PGD, but more importantly provides the basis for carrier detection and inductive pre-marital counselling which has the potential to prevent this and other similar hereditary diseases in future generations. The identification of a single or limited number of founder mutations in the population would also provide the basis upon which an inductive or selective screening program could be conducted. This would also depend on the representative nature of the samples screened in this study. Given that the samples tested are the total experience of our metabolic screening laboratory over a 10 year period and that it receives all cases of suspected inborn errors of metabolism in Saudi Arabia, the sample can be considered to be representative.

Our study confirms that the previously reported R41Q mutation in Saudi patients is indeed the most common mutation. It accounts for 89% of the pathogenic 3HMG alleles in Saudi Arabia. The high incidence of R41Q (89%) suggests a common founder and is consistent with the demographic observation that the majority of patients come from a single ancient tribe in Saudi Arabia. This was confirmed by genotype analysis at the D1S2620 microsatellite marker on chromosome 1 (see Results). The majority of the patients reported in the literature with R41Q mutation are of Saudi origin [18]. A Czech patient [24] and a Turkish patient homozygous for R41Q together with an Italian patient heterozygous for this mutation have also been identified [18]. Both Turkey and Italy were subject to migration from the Arabian Peninsula during the Arabic invasion. As observed with Carbonic Anhydrase II deficiency [25], the likely hood of R41Q migrating from the Arabian Peninsula exists, however the presence of this mutation in three different countries raises the hypothesis that this mutation has arisen independently more than once. More extensive haplotype analysis from patients from these regions may be helpful in tracing the origin of this mutation.

Among the 6 Saudi patients previously reported with this mutation, clinical data is available on four [26]. In two, symptoms started at 3 days of age and the other two at 2 and 6 months respectively. Of three patients included in our study from whom clinical data is available, two had onset of disease at 5 months and one at 3 days of age respectively. Furthermore, we identified six more patients homozygous for R41Q mutation in whom testing was offered on clinical grounds. All patients presented between 3–8 months of age following intercurrent illness. Currently all nine patients are on a restricted leucine and fat diet in addition to carnitine. They are followed at our metabolic clinic and are doing well with normal growth and development. The above data indicates that the R41Q mutation can be associated with either neonatal or infantile presentation. Furthermore, in contrast to previous suggestion that 3HMG in Saudi Arabia is a neonatal-onset organic acidemia [26], our data as shown above indicates that infantile presentation is also quite common. Good neurodevelopmental outcome is observed in the majority of the patents provided appropriate medical attention is given during episodes of crisis especially in the first few years of life.

No clinical data is available on the other two mutations identified in our study, however one Saudi patient reported previously with F305fs (-2) mutation had a neonatal onset of the disease. Growth and development was reported to be normal at 15 months of age [27]. It is interesting to note that this mutation has not been identified so far in any other ethnic group.

## Conclusion

Our study provides the most extensive genotype analysis on 3HMG patients from Saudi Arabia. Based on our results, we recommend that all patients of Saudi ethnicity diagnosed with 3HMG be tested for the above three mutations. Patients negative for these mutations should be the basis of further *HMGCL *sequence analysis to identify additional rare mutation(s) in the Saudi Population. Testing for these three mutations should also be included in any population based screening program for 3HMG in Saudi Arabia.

## Competing interests

The author(s) declare that they have no competing interests.

## Authors' contributions

MDS was responsible for the concept of the study, had a leading role in its design and coordination and drafted the manuscript. FI and OS carried out the molecular genetic studies. MR carried out the biochemical analysis and provided the appropriate DBS samples to the molecular lab. BM contributed to design, troubleshooting with the molecular analysis and revising the manuscript. All authors read and approved the final manuscript.

## Pre-publication history

The pre-publication history for this paper can be accessed here:


